# People and Pets in the COVID-19 Pandemic and the Cost-of-Living Crisis: Identifying Trends in the Intake, Adoption and Return of Companion Animals During Times of Uncertainty

**DOI:** 10.3390/ani15111584

**Published:** 2025-05-29

**Authors:** Lindsay Murray, Janine Carroll, Jane Tyson

**Affiliations:** 1 Division of Psychology, University of Chester, Chester CH1 4BJ, UK; j.carroll@chester.ac.uk; 2 Royal Society for the Prevention of Cruelty to Animals, Parkside, Chart Way, Horsham RH12 1GY, UK; jane.tyson@rspca.org.uk

**Keywords:** pet, rehoming, relinquishment, RSPCA, human–animal interaction, length of stay, interrupted time series analysis

## Abstract

Pet ownership is the most common form of human–animal interaction (HAI), is prevalent worldwide and confers benefits for the health and wellbeing of humans. Here, we examined a large set of anonymised data from the Royal Society for the Prevention of Cruelty to Animals (RSPCA) to identify trends in the intake, adoption and relinquishment of companion animals in England and Wales before and during times of uncertainty, namely, the COVID-19 pandemic and the cost-of-living crisis (COLC). We employed an interrupted time series (ITS) design which controls for pre-existing trends by comparing observed outcomes post-intervention with those expected if the intervention had not occurred. Nearly 200,000 animals were taken in by the RSPCA and over 140,000 animals were adopted or released over the four-year period from 2018 to 2022. When controlling for the brief closure of RSPCA sites, fewer dogs and cats were taken in and adopted during the pandemic compared to pre-pandemic, and the intake and adoption of animals were lower during the COLC than before. A downward trend in the return of animals was observed, and the principal reasons for return were problem behaviours, owner unpreparedness and a change in circumstances. Our ITS analysis also permitted forecast predictions to be made which could prove helpful to the RSPCA.

## Simple Summary

Pet ownership is the most common form of human–animal interaction, is prevalent worldwide and confers benefits for the health and wellbeing of humans. Here, we examined a large set of data from the Royal Society for the Prevention of Cruelty to Animals (RSPCA) to identify trends in the intake, adoption and return of companion animals in England and Wales before and during times of uncertainty, namely, the COVID-19 pandemic and the cost-of-living crisis (COLC). We used an interrupted time series analysis which controls for pre-existing trends by comparing observed outcomes post-intervention with those expected if the intervention had not occurred. Nearly 200,000 animals were taken in by the RSPCA and over 140,000 animals were adopted or released over the four-year period from 2018 to 2022. When controlling for the brief closure of RSPCA sites, fewer dogs and cats were taken in and adopted during the pandemic compared to pre-pandemic, and the intake and adoption of animals were lower during the COLC than before. A downward trend in the return of animals was observed, and the principal reasons for return were problem behaviours, owner unpreparedness and a change in circumstances. Our ITS analysis also permitted forecast predictions to be made which could prove helpful to the RSPCA.

## 1. Introduction

The study of human–animal interaction (HAI) is a developing field of research examining the relationship between people and animals and the effects that inter-species interactions have on both the psychological and physical health of people and animals. Pet ownership is the most common form of HAI with close to a billion pets keptworldwide [1]. Recent estimates from the People’s Dispensary for Sick Animals (PDSA) working with YouGov (a global public opinion and data company) show that 51% of adults in the UK own a pet, with 28% adults owning 10.6 million dogs combined and 24% having 10.8 million cats, more often living in multiple-cat households [2]. The estimated population of pet rabbits was 800,000. These organisations have tracked changing trends in pet ownership since 2011 with a particular focus on welfare [3]. Statistics such as these, together with insights from academic research, can best inform welfare initiatives and policy change such as the Animal Welfare (Import of Dogs, Cats and Ferrets) Bill.

Pet ownership is often linked to benefits for the health and wellbeing of humans, with pets providing comfort, safety, routine, structure, help with social inclusion and playing a meaningful role in people’s lives [4]. Some benefits are as yet identified only in particular groups of animals; for example, dog owners fall asleep more easily than non-owners [5]. Particular groups of people may also benefit; for example, the immunosuppressed, with a tendency towards a more sedentary lifestyle, show less of a decline in physical activity and increased sleep when they have a dog [6]. An interesting review of so-called companion animals, utilising the Tinbergian ethological framework [7], argues that only cats and dogs can be considered as ‘true’ companion animals, because their evolutionary past led them to not only associate with humans (as do other domesticated species) but show a preference to stay with them. Other species, with reduced interactions with humans, may be better described as ‘animated collectibles’ (e.g., exotic birds) or ‘captive pets’ (e.g., other domesticated species such as horses) [8]. The strength with which cats and dogs can be attracted to and bonded with their human owners often results in them being regarded as more like family members than pets.

However, a recent review has shown that the impact on physical and mental health is difficult to determine due to inconsistencies in study design, and that not all aspects of pet ownership are positive [9]. In terms of global biodiversity and climate crises, keeping pets is not without impact, as they have their own carbon ‘pawprint’ just as humans have a carbon footprint and are significant consumers and producers of waste. As our climate continues to change, the importance of research into crisis-driven changes in human–animal relationships increases.

Humans can be vulnerable to change and particularly stressful times of uncertainty such as the pandemic and the cost-of-living crisis (COLC) can easily disrupt lifestyle behaviours [10]. For example, during the coronavirus pandemic, global interest in pet ownership (as measured by internet search volume) increased in the early stages and was sustained for some but not all types of pets once restrictions were lifted [11]. This could be because of the impact of rules around social distancing and the associated increase in loneliness. People were still able to walk their dogs while there were restrictions [12]. which would likely contribute to a better quality of life for them in terms of physical activity, a change in scene and fresh air. The news media certainly gave the impression that pet adoptions were increasing during the pandemic [13]; however, the data, largely coming from the US, do not tend to support this picture [14,15]. The COLC could realistically push people to consider drastic decisions to give up their pets. Seventy-eight per cent of people felt that this crisis would have a negative effect on pet welfare [2], so, even if not relinquished, the conditions in which pets are kept may change for the worse.

Animals unfortunately are abandoned or mistreated daily. The hope is that most, if not all, of these will be successfully rehomed; however, following adoption, as many as 7% to 20% of animals are returned to shelters [16], a source of stress for both the animal and the owner. The reasons for returning a pet may include the owner’s health, unrealistic expectations of ownership duties, pet behavioural issues and incompatibility with existing pets [17]. It may be due to the wellbeing impact not being as positive as expected [18]. It has also been found that nearly two-thirds of relinquishers were first-time pet owners and that households with children were 4.6 times more likely to consider or have already given up their cat or dog [19]. After the aforementioned increased interest in pet ownership during the pandemic, the media again told of a subsequent rise in the abandonment of pets [20]. While some relinquishments may have occurred due to a lack of fit between the new pet companion and the owner returning to a workplace, or even due to fear of potential virus transmission [21], the data again do not support such a trend [22,23,24]. There is a need to analyse the trends in the adoption and return rates of pets throughout the pandemic to support the future welfare of animals and their owners. Identifying factors that influence pet owners’ decisions to acquire a pet, and whether these factors are prevalent in different species and in different circumstances, will help our understanding of the outcomes people experience when living with their pet. It may also allow interventions to be planned with the aim of optimising compatibility at the adoption stage and reducing the relinquishment of companion animals.

In a review of the factors associated with acquiring a dog, external influences, such as household structure, socioeconomic status, prior dog ownership and specific dog characteristics (e.g., breed, popularity, age), as well as individual and psychological factors, such as similarities in human and dog personalities and the temperament of the dog, all contributed to the decision-making process [25]. However, it is unclear whether these factors are applicable to all pets or just to dog acquisition. Furthermore, wider social and environmental conditions may also influence the acquisition of pets from animal shelters.

The breed and age of the pet have been found to predict the likelihood of return [16]. With vets severely limiting their practice during the pandemic, there were consequences for certain companion animals; for example, cats and rabbits were not neutered, resulting in population explosion and large numbers being rescued (RSPCA, personal discussion). Pets with behavioural problems, such as destructive behaviour, soiling and vocalising, account for a large proportion of returned animals. Pets with such issues risk abandonment or euthanasia if not relinquishment to a shelter [26]. The impact on owners can be considerable with more time and effort needed to manage, tackle and train the pet, difficulties in exercising dogs, limitations on visitors and strains on family relationships [27]. Just as living with a family member who is ill impacts the whole family [28], the same can be true with a nonhuman family member, especially if the issue is chronic.

Having any pet, or multiple pets, incurs an obvious financial cost, including for food, accessories and vet fees. It would be expected that the COLC would likely result in people making the decision to reduce their household costs by parting with some or all of their companion animals. According to the Institute for Government and the UK Office of National Statistics, the cost-of-living crisis began in October 2021, when people felt a dramatic fall in their disposable income due to increases in inflation and taxes, disruptions to global supply chains as a result of the pandemic and, in particular, the rise in energy costs following the war in Ukraine. Although the government responded to the crisis by offering packages of support and an Energy Price Guarantee, it is unlikely that people at the extreme end of financial suffering could prioritise keeping pets. The price of essential goods is increasing at a rate faster than household incomes, which are not expected to return to 2021 levels before 2027. Research is beginning to show the varied effects of food and fuel poverty, from experiencing greater food insecurity and a poorer quality diet [29], to the incidence of hot water bottle burns increasing as people try to reduce central heating [30].

Our study adds to the growing literature on this aspect of human–animal relationships by including early responses to the COLC and by including animal shelters throughout England and Wales. We examined a large set of anonymised data from the Royal Society for the Prevention of Cruelty to Animals (RSPCA) to identify trends in the intake, adoption and relinquishment of companion animals in England and Wales before and during times of uncertainty, namely, the COVID-19 pandemic and the cost-of-living crisis. The RSPCA has 14 national centres that take in companion animals, mainly through the work of their inspectorate, alongside a network of 134 branches, which are independently registered charities operating separately from the national centres. Of these, 45 have an animal centre which varies between sites in terms of facilities and capacities; other branches rehome animals through external connections (e.g., private boarding establishments, foster homes). Whilst branches take animals from the inspectorate, the figure is much lower than national centres, at approximately 35% of their overall intake; the remaining 65% of animal intake is due to a variety of reasons, including welfare risk, returns, wildlife and abandoned animals not stemming from inspectorate work. The RSPCA takes in sick/injured stray animals, but not stray dogs who fall under the remit of local authorities, nor do they take in healthy stray cats. When breaking down rehoming between national centres and branches, approximately 80% of rehoming is conducted by branches with the remaining 20% conducted at national centres.

With data covering the period 2018–2022, we analysed the overall pattern of trends over time in intake, adoption and returns across RSPCA centres and branches, both separately and combined. We predicted the following:

1. During the pandemic, more animals would be taken in and/or returned because of concerns about the spread of the virus or pressures created in work–life balance due to people working at home more;
2. During the pandemic, more animals would be adopted for reasons of companionship and leisure;
3. During the pandemic, more wildlife would be taken in and rehabilitated because people would be encountering them while out walking in nature more than they had previously;
4. Following the pandemic, more animals would be taken in and/or returned because of the change in circumstances as people returned to work outside of the home;
5. Following the pandemic, fewer animals would be adopted due to concerns about virus transmission;
6. The intake and return of animals would be higher, and adoptions lower, during the cost-of-living crisis compared to before the crisis due to people feeling unable to cope with the extra expense of caring for pets.

## 2. Method

### 2.1. Data Sources

We obtained data on the number of animals taken in by, adopted from and returned to the RSPCA from December 2018 to November 2022. Due to the different ways in which data were recorded at the animal centres and the branches, we combined the data where possible but also examined these two groups separately. Individual-level data were aggregated into monthly sums, and means were calculated for the different species recorded. Some data were available for the length of stay of animals in shelters, and this was calculated from the date of arrival to the date of rehoming. When animals were returned to the RSPCA, owners were asked to provide the main reason for this return. This study received approval from the RSPCA and from the Division of Psychology Ethics Committee at the University of Chester.

### 2.2. Study Design

We employed an interrupted time series (ITS) design to evaluate the impact of both the COVID-19 pandemic and the cost-of-living crisis (COLC) on the intake, adoption and return of animals over time. The ITS controls for pre-existing trends by comparing observed outcomes post-intervention with those expected if the intervention had not occurred. This is a useful analytical tool to use when full randomisation is not possible and is more cost-effective and often quicker than randomised control trials [31,32]. In this way, our study acts like a quasi-experiment and enables us to determine the impact of COVID-19 pandemic lockdowns and uncover whether any effects have been sustained since. In regard to the COLC, we compare the periods before and during the crisis. The ITS also permits predictions to be made which can be useful in many economics and business industries and could prove helpful to the RSPCA. We tested both exponential smoothing models (for seasonality) and ARIMA forecasting models, with the latter being one of the two most popular methods for the ITS regression model [33], which takes into consideration the key parameters of trend, seasonality, non-stationarity and autocorrelation. The AR in ARIMA stands for autoregressive and refers to the ‘memory’ of how past values tend to affect future values; the I refers to integrated (differencing) components and accounts for systematic changes in slope and variance (for example, trends and seasonality); and the MA stands for the moving average component, and this uses the ‘memory’ of past errors to smooth out the noise (variability) in the data by removing non-deterministic or random movements from the time series. These models are empirically based prediction models, where SPSS v.29 has taken out as much of the noise as possible due to the effects of seasonality, trend and autocorrelation and provides the model of best fit to the data.

### 2.3. Study Period, Intervention and Outcomes

In this ITS study, we defined the interventions as the pandemic and the COLC. Three time periods were identified for the pandemic: December 2018–March 2020 (pre-pandemic, *n* = 16 months), April 2020 through July 2021 (pandemic lockdowns and restrictions, *n* = 16 months) and from August 2021 to November 2022 (post-pandemic, *n* = 16 months). This constitutes a very good sample size, having 16 months on either side of the 16-month restriction period, and coincides with the median number of time points and the modal time interval in a review of 153 ITS studies [33]. As the UK is still currently experiencing the COLC, we used a straightforward before and during analysis to examine trends in the intake, adoption and return of animals, using October 2021 as the beginning of the crisis. We also included closure as a covariate. Animal centres and branches were closed to the public during lockdown, but essential services such as rehoming continued to run remotely. Potential adopters could apply for animals online via the ‘Find a Pet’ site and were assessed via discussions, videos and photos of their homes and virtual home visits. Adopted animals were delivered to their new homes following social distancing guidance. Vets ran emergency clinics only, so neutering and routine treatments were often unavailable to clients. The outcomes of interest were the monthly totals and means of intakes, adoptions and returns of animals over time.

### 2.4. Statistical Methods

Descriptive statistics were computed and data from centres and branches were analysed separately using one-way Anovas (pandemic) and independent *t* tests (COLC) to test for differences over time. We then used sequence charts to visually inspect the combined data from both centres and branches for each animal category to determine any underlying trends or seasonal patterns, as well as any outliers. Interrupted time series analyses [34] based on regression were used to consider the pattern of intake, adoption and return numbers over time. The linear regression model used in ITS analysis can highlight trends in the rates of the intake, adoption and return of animals, as well as uncover seasonal variation [35]. The model equations use the standard Ot for the measurement of outcome indexed over time (t), which here are the aggregated numbers of animals taken in by, adopted from and returned to the RSPCA each month. X is used to denote the interruption, which here would be the pandemic with three levels—pre-pandemic, during the pandemic and post-pandemic—and the COLC with two levels: pre-COLC and during COLC. The intervention effects were modelled using dummy variables dichotomously coded for 0 representing pre- and post-pandemic and 1 for pandemic, and 0 for pre-COLC and 1 for during COLC. Due to some of the RSPCA sites being closed during particular lockdown periods, closure was included as a covariate with months involving closure disruption coded as 1 versus normal months coded as 0. Tests were one-sided where predictions were made with *p* values less than 0.05 considered statistically significant.

## 3. Results

### 3.1. Animal Intake by RSPCA over Time

**Figure 1:**
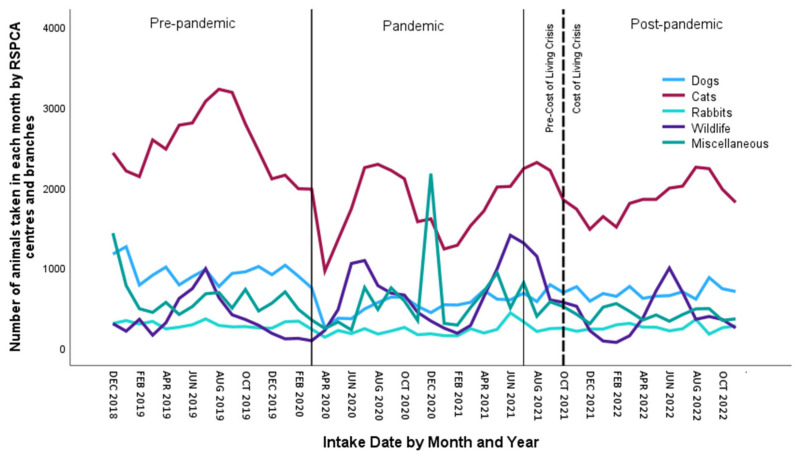
Number of animals taken in each month by RSPCA centres and branches during the three time periods of the pandemic and the two time periods of the COLC.

Overall, between December 2018 and November 2022, 198,545 animals were taken in by the RSPCA. At centres, these were recorded as dogs, cats, rabbits, other companion animals, wildlife and equines, while at branches, they were categorised as dogs, cats, rabbits, miscellaneous animals and wildlife. The ‘other’ and ‘miscellaneous’ categories included pet rodents, ferrets, fish, exotics and captive birds; for consistency, we will refer to both of these categories as ‘miscellaneous’. Figure 1 shows the total number of animals taken in each month by RSPCA centres and branches during the three time periods related to the pandemic and the two time periods related to the COLC. Table 1 displays the means and standard deviations for the same time periods related to these two major events.

Figure 1 shows no clear trend across time for any animal type. When looking at cats, we can see evidence of some autocorrelation, meaning that points closer together tend to be more similar to each other than they are to points further apart. It also appears that cat intake peaks during the summer months of July–August in all years which shows seasonality. Wildlife intake also peaks during the summer.

(a) Animal Centres
  (i) COVID-19 Pandemic

One-way Anovas showed that there were significant differences in the numbers of animals taken in by RSPCA centres depending on the pandemic status for all categories except miscellaneous animals, with *F*(2,45) = 1.64, *p* = 0.103 and *η*^2^ = 0.068—that is, for dogs, *F*(2,45) = 20.22, *p* < 0.001 and *η*^2^ = 0.473; for cats, *F*(2,45) = 19.58, *p* < 0.001 and *η*^2^ = 0.465; for rabbits, *F*(2,45) = 4.92, *p* = 0.006 and *η*^2^ = 0.179; for equines, *F*(2,45) = 5.63, *p* = 0.004 and *η*^2^ = 0.200; and for wildlife, *F*(2,45) = 3.04, *p* = 0.029 and *η*^2^ = 0.119. Although not significant overall, a clear spike in the intake of miscellaneous animals is evident for the period around Christmas of 2020 (Figure 1). This reflects an occurrence where a larger number (in this case, 1663) of animals had to be taken in at one point in time. As this reportedly happens from time to time, we did not consider it an outlier. Post hoc Tukey tests uncovered that significantly fewer dogs, cats and rabbits were taken in during the pandemic compared to before (all *p* < 0.001). Following the pandemic, significantly more dogs (*p* = 0.025) and rabbits (*p* = 0.013) were taken in compared to during the pandemic, but most of the other animal categories showed a downward trend in intake post-pandemic. For dogs (*p* < 0.001), cats (*p* < 0.001), equines (*p* = 0.003) and wildlife (*p* = 0.023), significantly fewer animals were taken in post-pandemic compared to pre-pandemic.

     (ii) Cost-of-Living Crisis

Contrary to our prediction, the numbers of animals taken in by centres during the COLC were actually lower than prior to the crisis for all animal types except rabbits (pre-COLC mean 250.24; COLC mean 252.21, *t*(46) = −0.10, *p* = 0.461). These trends were significant for cats (pre-COLC mean 2146.09; COLC mean 1855.86, *t*(45.94) = 2.57, *p* = 0.007), equines (pre-COLC mean 56.88; COLC mean 31.29, *t*(46) = 1.92, *p* = 0.031) and miscellaneous animals (pre-COLC mean 607.09; COLC mean 423.57, *t*(39.72) = 2.80, *p* = 0.004), but not for dogs (pre-COLC mean 731.74; COLC mean 689.43, *t*(44.60) = 0.922, *p* = 0.181) or wildlife (pre-COLC mean 540.03; COLC mean 408.71, *t*(46) = 1.21, *p* = 0.116).

(b) RSPCA Branches
  (i) COVID-19 Pandemic

One-way Anovas showed that there were significant differences in the numbers of animals taken in by RSPCA branches depending on the pandemic status for all categories except miscellaneous animals, with *F*(2,45) = 1.38, *p* = 0.131 and *η*^2^ = 0.058—that is, for dogs, *F*(2,45) = 45.84, *p* < 0.001 and *η*^2^ = 0.671; for cats, *F*(2,45) = 16.86, *p* < 0.001 and *η*^2^ = 0.428; for rabbits, *F*(2,45) = 6.80, *p* = 0.002 and *η*^2^ = 0.232; and for wildlife, *F*(2,45) = 4.20, *p* = 0.011 and *η*^2^ = 0.157. For dogs (*p* < 0.001), cats (*p* < 0.001) and rabbits (*p* = 0.003), significantly fewer animals were taken in during the pandemic. Post-pandemic, numbers rose again for dogs (*p* = 0.001), but not to pre-pandemic levels. For cats and rabbits, the rise in numbers post-pandemic was not significantly different to pandemic levels. Wildlife showed a distinctly opposite pattern with more animals being taken in during the pandemic (*p* = 0.008), and while this reduced post-pandemic, it remained higher than at pre-pandemic levels, although not significantly so. There was a downward trend in the intake of miscellaneous animals.

     (ii) Cost-of-Living Crisis

Contrary to our prediction, and similar to the pattern in centres, the numbers of animals taken in by branches during the COLC were lower than prior to the crisis for all animal types. These trends were significant for cats (pre-COLC mean 1681.56; COLC mean 1477.00, *t*(45.57) = 2.35, *p* = 0.006) and rabbits (pre-COLC mean 185.38; COLC mean 166.93, *t*(42.50) = 1.83, *p* = 0.019), but not for miscellaneous animals (pre-COLC mean 352.65; COLC mean 290.14, *t*(46) = 1.13, *p* = 0.067), dogs (pre-COLC mean 521.38; COLC mean 488.86, *t*(40.04) = 1.01, *p* = 0.075) or wildlife (pre-COLC mean 513.88; COLC mean 404.07, *t*(46) = 1.02, *p* = 0.075).

We then examined both the pandemic and the COLC in an interrupted time series model. As can be seen in Figure 1, the patterns of intake over time are changeable. This makes the data appropriate for a time series analysis which has stationarity as an assumption as opposed to trending data.

Simple seasonal models were the best fit for rabbit (*R*^2^ = 0.676, *p* < 0.001), wildlife (*R*^2^ = 0.616, *p* < 0.001) and miscellaneous animal (*R*^2^ = 0.852, *p* < 0.001) intake, while a Winters’ Additive model fit the equine data better (*R*^2^ = 0.871, *p* < 0.001). ARIMA models were better for dogs (1,1,0)(0,0,0) and cats (0,1,3)(0,1,0) (Figure 1). Taking dogs as an example, the numbers in brackets relate to the following parameters: 1 refers to autoregression (AR), the middle 1 refers to first differencing (simple upward trend for component I), and the final 0 refers to the moving average (MA) component. The closure of RSPCA sites significantly reduced the intake of dogs, with 157 fewer dogs per month (*R*^2^ = 0.429, *p* < 0.001) being taken in compared to when sites were open. The pandemic resulted in 764 fewer cats (*R*^2^ = 0.586, *p* < 0.001) and 507 fewer dogs (*R*^2^ = 0.429, *p* < 0.001) per month being taken in compared to pre-pandemic levels. The COLC had no effect in the time series analysis. Table 2 below shows that the pandemic was responsible for significant reductions in dogs and cats being taken in, while the closure of RSPCA sites was responsible for fewer dogs being taken in.

**Figure 2:**
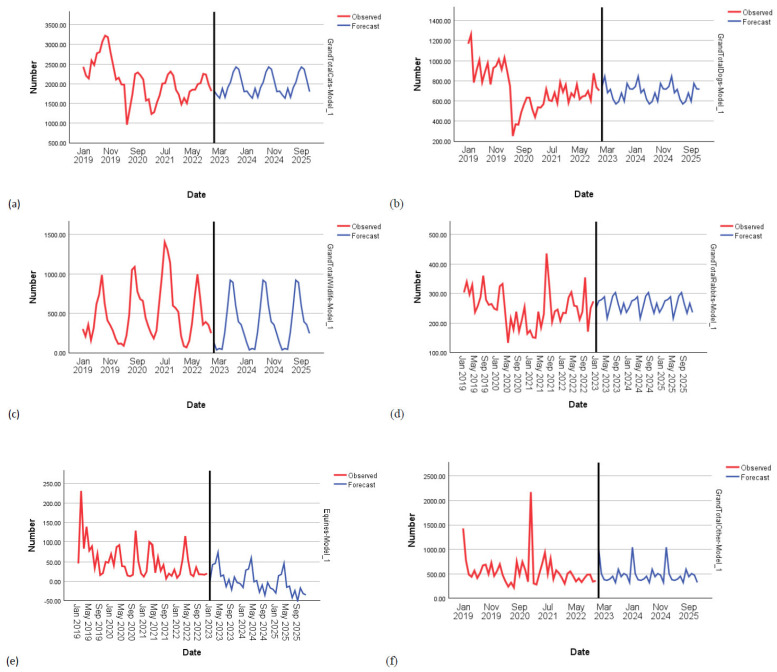
Intake of animals from 2018 to 2022, with forecasted predictions to 2025 for (**a**) cats, (**b**) dogs, (**c**) wildlife, (**d**) rabbits, (**e**) equines and (**f**) miscellaneous animals.

Figure 2 shows the trends in the intake data for each animal type together with predictions forecasted to 2025.

### 3.2. Animal Adoptions from the RSPCA over Time

**Figure 3:**
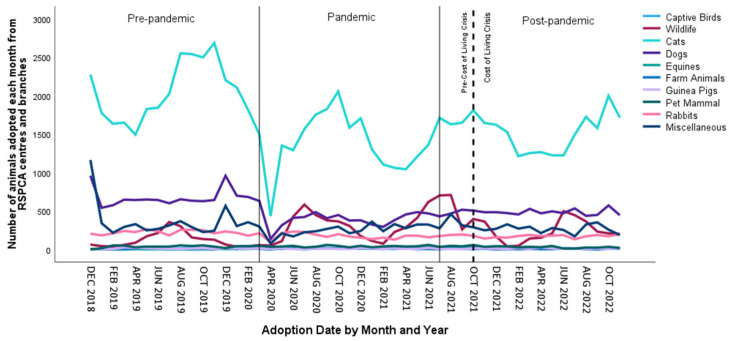
Number of animals adopted from RSPCA centres and branches during the three time periods of the pandemic and the two time periods of the COLC.

In total, 142,737 animals were adopted from the RSPCA over the four years, recorded as dogs, cats, rabbits, pet mammals, guinea pigs, captive birds, farm animals and equines at the centres, and as dogs, cats, rabbits, miscellaneous animals and wildlife at the branches. We include wildlife here, but it should be noted that wildlife are not rehomed but rather rehabilitated and released back into the wild. Figure 3 shows the total adoptions of animals from the RSPCA over time. For cats, dogs and rabbits, this is a combined total from both centres and branches; for the remaining categories, the figures are from centres or branches only. The peaks for cat adoptions occur during the autumn months, which corresponds to the intake during the preceding summer months.

Table 3 below shows clearly that adoptions of dogs, cats and rabbits fell in the pandemic but started to rise again post-pandemic, but not yet to pre-pandemic levels. The COLC may have influenced the reduction in adoptions of these animals too.

(a) Animal Centres
  (i) COVID-19 Pandemic

One-way Anovas showed that there were significant differences in the numbers of animals adopted from RSPCA centres depending on the pandemic status, with *F*(2,45) = 9.51, *p* < 0.001 and *η*^2^ = 0.297 for cats, *F*(2,45) = 4.45, *p* = 0.009 and *η*^2^ = 0.165 for rabbits, and *F*(2,45) = 22.10, *p* < 0.001 and *η*^2^ = 0.496 for equines, with all other groups being not significant. Post hoc Tukey tests uncovered that significantly fewer cats were adopted during the pandemic compared to before (*p* < 0.001). However, significantly more equines were adopted during the pandemic compared to before (*p* < 0.001). Significantly fewer cats (*p* = 0.001) and rabbits (*p* = 0.007) were adopted post-pandemic compared to pre-pandemic (*p* < 0.001), while equines were adopted more post-pandemic compared to before (*p* < 0.001).

     (ii) Cost-of-Living Crisis

Supporting our prediction, and in contrast to the pattern with intakes, the numbers of animals adopted from centres during the COLC were significantly lower than prior to the crisis for most animal types: cats (pre-COLC mean 241.94; COLC mean 195.92, *t*(45.96) = 1.68, *p* = 0.005), rabbits (pre-COLC mean 34.94; COLC mean 26.00, *t*(46) = 1.96, *p* = 0.025), pet mammals (pre-COLC mean 38.37; COLC mean 27.69, *t*(46) = 2.74, *p* = 0.002) and farm animals (pre-COLC mean 7.57; COLC mean 4.85, *t*(46) = 1.74, *p* = 0.022). These reductions were not significant for captive birds (pre-COLC mean 12.34; COLC mean 10.23, *t*(46) = 0.84, *p* = 0.102) or dogs (pre-COLC mean 76.06; COLC mean 74.38, *t*(43.00) = 0.28, *p* = 0.195). For equines (pre-COLC mean 10.77; COLC mean 15.92, *t*(46) = −1.67, *p* = 0.025) and guinea pigs (pre-COLC mean 9.66; COLC mean 11.46, *t*(46) = −1.00, *p* = 0.070), there were more adoptions during the COLC.

(b) RSPCA Branches
  (i) COVID-19 Pandemic

One-way Anovas showed that there were significant differences in the number of animals adopted from RSPCA branches depending on the pandemic status for dogs, with *F*(2,45) = 38.75, *p* < 0.001 and *η*^2^ = 0.633, cats, with *F*(2,45) = 12.75, *p* < 0.001 and *η*^2^ = 0.362, rabbits, with *F*(2,45) = 16.66, *p* < 0.001 and *η*^2^ = 0.425, and miscellaneous animals, with *F*(2,45) = 2.55, *p* = 0.045 and *η*^2^ = 0.102. Significantly fewer dogs, cats and rabbits (all *p* < 0.001) were adopted during the pandemic compared to pre-pandemic, although this was not significant for miscellaneous animals. For wildlife, however, significantly more animals were released during the pandemic, with *F*(2,45) = 6.97, *p* = 0.001 and *η*^2^ = 0.236, and there was a significant increase post-pandemic compared to before (*p* = 0.013). Significantly fewer dogs (*p* < 0.001), cats (*p* = 0.001) and rabbits (*p* < 0.001) were adopted post-pandemic compared to pre-pandemic (*p* < 0.001), while equines were adopted more post-pandemic compared to before (*p* < 0.001).

     (ii) Cost-of-Living Crisis

Supporting our prediction, and in contrast to the pattern with intakes, the numbers of animals adopted from branches during the COLC were significantly lower than prior to the crisis for dogs (pre-COLC mean 451.40; COLC mean 409.31, *t*(41.329) = 1.43, *p* = 0.040), cats (pre-COLC mean 1468.49; COLC mean 1303.08, *t*(46) = 1.36, *p* = 0.045) and rabbits (pre-COLC mean 160.06; COLC mean 143.54, *t*(40.482) = 2.42, *p* = 0.005), but not for miscellaneous animals (pre-COLC mean 311.49; COLC mean 264.23, *t*(46) = 0.980, *p* = 0.083) or wildlife (pre-COLC mean 244.03; COLC mean 235.54, *t*(46) = 0.141, *p* = 0.222).

We then examined both the pandemic and the COLC in an interrupted time series model, along with closure as a covariate. As can be seen in Figure 3, the patterns of adoption over time are also changeable.

Simple seasonal models were the best fit for pet mammal (*R*^2^ = 0.766, *p* < 0.001), guinea pig (*R*^2^ = 0.805, *p* < 0.001), captive bird (*R*^2^ = 0.824, *p* < 0.001) and equine (*R*^2^ = 0.665, *p* < 0.001) adoptions, and for wildlife release (*R*^2^ = 0.704, *p* < 0.001). ARIMA models were better for dogs (0,1,0)(0,0,0), cats (0,1,0)(1,0,0), rabbits (0,1,1)(0,0,0) and farm animals (0,0,0)(0,0,1). For miscellaneous animals, a Winters’ Multiplicative model was the best fit (*R*^2^ = 0.624, *p* < 0.001) (Figure 3). The closure of RSPCA sites significantly reduced the adoption of animals, with 520 fewer cats per month (*R*^2^ = 0.484, *p* < 0.001) and 57 fewer rabbits (*R*^2^ = 0.388, *p* < 0.001) per month. The pandemic significantly impacted the adoption of animals, with 997 fewer cats (*R*^2^ = 0.484, *p* < 0.001) and 494 fewer dogs (*R*^2^ = 0.326, *p* < 0.001) being adopted each month. Table 4 below shows that the pandemic and the closure of the RSPCA were responsible for significant changes in the adoption of animals.

**Figure 4:**
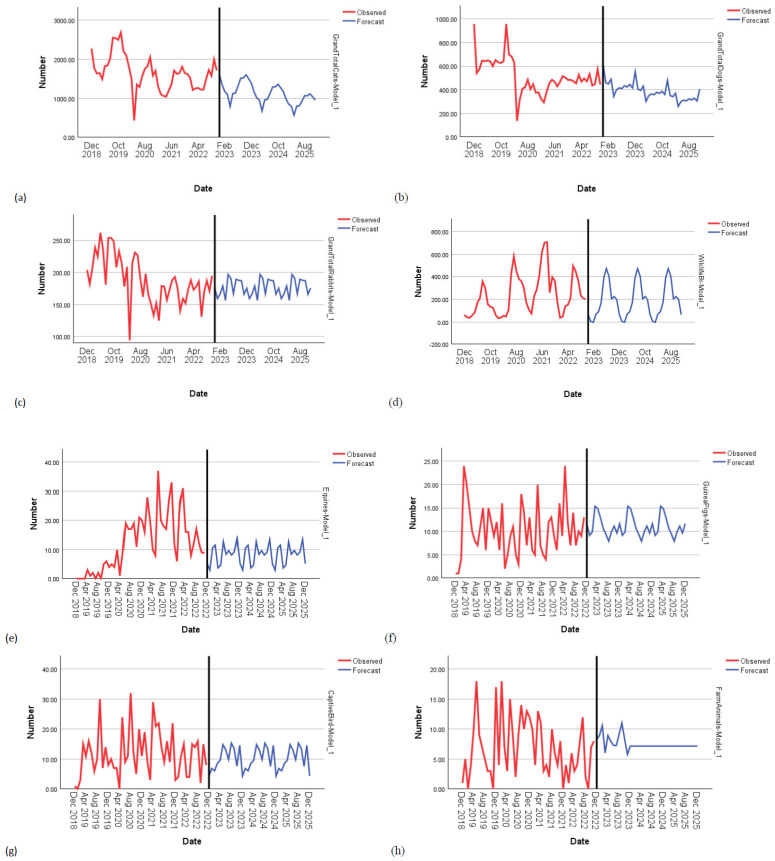

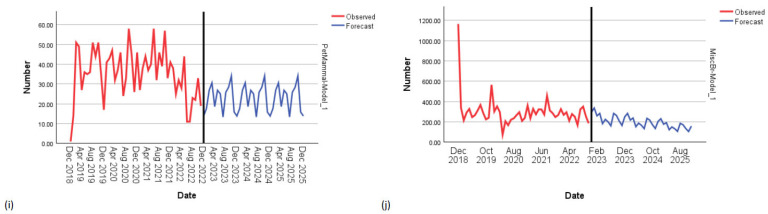
Adoption of animals from 2018 to 2022, with forecasted predictions to 2025 for (**a**) cats, (**b**) dogs, (**c**) rabbits, (**d**) wildlife release, (**e**) equines, (**f**) guinea pigs, (**g**) captive birds, (**h**) farm animals, (**i**) pet mammals and (**j**) miscellaneous animals.

Figure 4 shows the trends in the adoption data for each animal type together with predictions forecasted to 2025.

*a.*  *Length of Stay before Adoption*

We also examined adoption by the length of stay for cats (Table 5), dogs (Table 6) and rabbits (Table 7).

Table 5 shows that cats stayed waiting to be rehomed for longer during the pandemic than pre-pandemic (*p* < 0.001) and longer again post-pandemic (*p* < 0.001). One-way Anova showed these differences to be significant (*F*(2) = 146.35, *p* < 0.001, *η*^2^ = 0.024). It is noteworthy that the length of stay increased despite the number of cats decreasing.

Table 6 shows that dogs stayed waiting to be rehomed for longer during the pandemic than pre-pandemic (*p* < 0.001), but in contrast to the picture with cats, the length of stay returned to pre-pandemic levels in the post-pandemic period (*p* < 0.001). One-way Anova showed these differences to be significant (*F*(2) = 9.944, *p* < 0.001, *η*^2^ = 0.005).

Table 7 shows that rabbits stayed waiting to be rehomed for longer during the pandemic than pre-pandemic (*p* < 0.001) and longer again post-pandemic (*p* < 0.001). One-way Anova showed these differences to be significant (*F*(2) = 102.047, *p* < 0.001, *η*^2^ = 0.109). It is noteworthy that the length of stay increased despite the number of rabbits decreasing.

### 3.3. Animals Returned to RSPCA over Time

**Figure 5:**
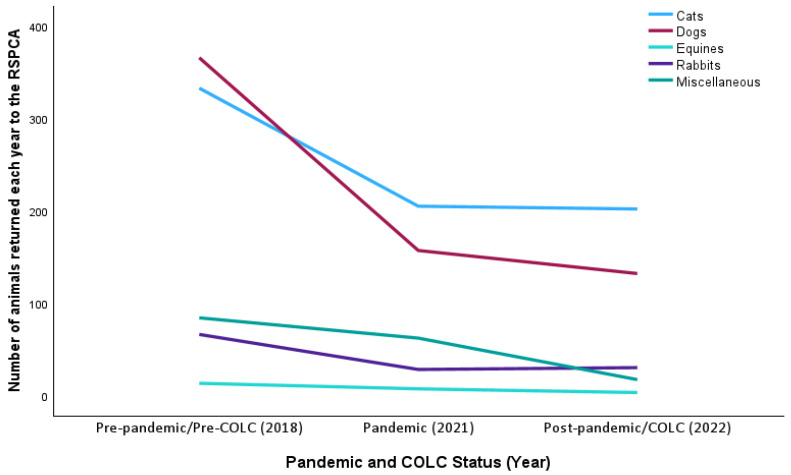
Number of animals returned to the RSPCA in each year.

The data provided by the RSPCA for returns were coded in a different way, not separated into centres and branches, and recorded purely by year. Unfortunately, due to a system change in record-keeping, there were no return data available for 2019 or 2020. So, for return data only, we decided to code the year 2018 as pre-pandemic, 2021 as pandemic and 2022 as post-pandemic. For the COLC, we compared 2022 with 2018. Returns were recorded for cats, dogs, equines, rabbits and miscellaneous animals (Figure 5).

    (i) COVID-19 Pandemic

Figure 3 shows a downward trend in returns for all animal types except rabbits, for which returns dropped during the pandemic but rose again post-pandemic.

    (ii) Cost-of-Living Crisis

Through a comparison of the data from 2018, representing before the COLC, with 2022, during the crisis, a similar downward trend in the return of all animal types is clear.

As the numbers were smaller and restricted to records by year only, we decided not to run analyses of variance and the ITS on the return data. However, we examined the data in more detail in terms of time after adoption and reasons for returns. Table 8 shows that for all animal types, fewer animals were returned within six months of having been adopted.

When animals were returned to the RSPCA, a record was made of the reasons, and these were categorised into the following: aggression, behavioural traits, change in financial position/environment, owner allergy, and unprepared for commitment. There was also an unknown category and the following categories, which we combined into ‘other’: centre management discretion, elective intake, stray dog, cat or equine and RSPCA-generated. Temporary boarding for equines and an ’unowned sick/injured’ category for miscellaneous animals (both having counts < 5) were not included as they were deemed not to be returned animals.

**Figure 6:**
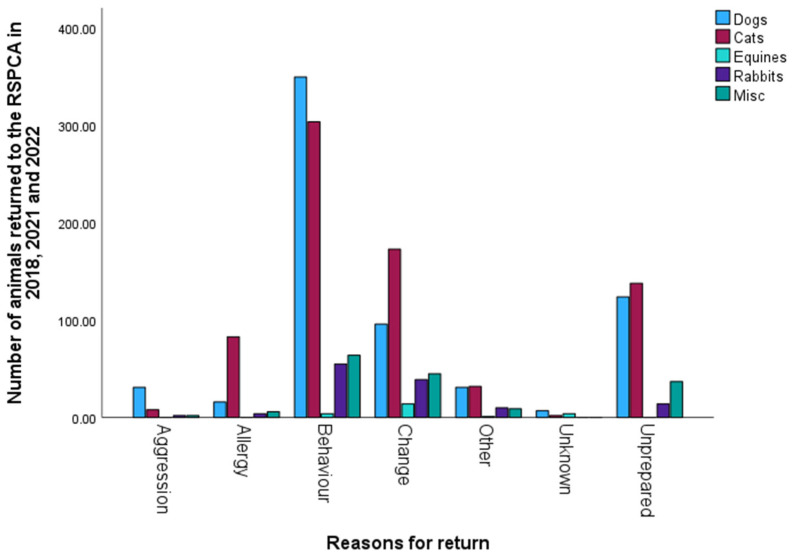
Reasons for the return of animals to the RSPCA in each year.

Significant associations were found between each animal type and the reasons they were returned to the RSPCA (Figure 6). Dogs were more likely to be returned due to behaviour problems, followed by owners being unprepared for the commitment and a change in circumstances (χ^2^(30) = 3275, *n* = 655, *p* < 0.001). Cats were returned more often due to behavioural issues, a change in circumstances and owners being unprepared (χ^2^(36) = 4440, *n* = 740, *p* < 0.001). For equines, most returns were due to a change in circumstances (χ^2^(6) = 46, *n* = 23, *p* < 0.001). Rabbits were returned more often due to behavioural issues and a change in circumstances (χ^2^(25) = 620, *n* = 124, *p* < 0.001). Behavioural issues, a change in circumstances and owners being unprepared were also the main reasons why miscellaneous animals were returned (χ^2^(25) = 815, *n* = 163, *p* < 0.001).

## 4. Discussion

### 4.1. Animal Intake

Nearly 200,000 animals were taken in by the RSPCA over the four-year period, translating to an average of 50,000 per year. Intake peaks during the summer months were found for cats, confirming the known ‘kitten season’. Wildlife intake also peaked in the summer months, with RSPCA centre staff reporting that this peak in admissions between May and August tends to be due to orphans, nestlings and fledglings, but also because people are outside more and more likely to come across wildlife casualties.

While fewer dogs, cats and rabbits were taken in during the pandemic by centres and branches compared to pre-pandemic, wildlife showed a distinctly opposite pattern with more animals being taken in during the pandemic, thus supporting our third prediction, that more wildlife would be taken in and rehabilitated because people would be encountering them while out walking in nature more than they had previously. During the pandemic, there were many reports that wildlife had become more noticeable, perhaps due to the quietening of human life with far less traffic on the roads and people staying at home. Wild animals were likely venturing into the open a little more, but it is also likely that people were just noticing animals that they had not noticed before. While people were outdoors engaging in their allotted daily walks and observing nature more astutely, they may have discovered and reported wildlife that they saw in distress. The intake of miscellaneous animals also rose slightly during the pandemic.

Following the pandemic, more dogs, cats and rabbits were taken in compared to during the pandemic, but numbers had not equalled those of pre-pandemic times. This supports our fourth prediction that following the pandemic, more animals would be taken in and/or returned because of the change in circumstances as people returned to work outside of the home. Wildlife and miscellaneous animals were taken in in smaller numbers post-pandemic compared to during the pandemic.

Overall, our interrupted time series (ITS) analysis revealed that the closure of RSPCA sites meant that fewer dogs were taken in compared to when sites were open; however, even when controlling for closure, fewer dogs and cats were taken in during the pandemic compared to pre-pandemic, therefore not supporting our first prediction, that during the pandemic, more animals would be taken in and/or returned because of concerns about the spread of the virus or pressures created in work–life balance due to people working at home more. Going against our final prediction, the intake of animals was actually lower during the COLC than before for dogs, cats, wildlife, equines and miscellaneous animals, and the COLC had no overall effect in the ITS analysis. Although the closure of sites may have prevented some animals in need from being cared for, the fact that fewer animals generally were being taken in by the RSPCA in the earlier stages of the ongoing COLC is encouraging as it suggests that people were not feeling forced into reducing household costs through surrendering their pets.

### 4.2. Animal Adoption and Length of Stay

Over 140,000 animals were adopted or released (in the case of wildlife) over the four-year period, resulting in an average of over 35,000 per year. Therefore, more animals were taken in than adopted each year. Overall, there were trends in the adoption of animals falling during the pandemic but then starting to rise again post-pandemic; thus, our fifth prediction, that following the pandemic, fewer animals would be adopted due to concerns about virus transmission [21], was not supported. The pattern for adoptions may have been affected by the concurrent fall in intake during this time; thus, both the intake and adoption of animals fluctuated in a similar manner. Cat adoptions peaked in the autumn, following the increased intake in the summer kitten season.

Our ITS analysis identified that the pandemic resulted in fewer dogs and cats being adopted, and a continued downward trend was forecast from the data. Therefore, our second prediction, that more animals would be adopted during the pandemic for reasons of companionship and leisure, was not supported, although more captive birds were adopted during the pandemic. The closure of sites resulted in fewer cats and rabbits being adopted. Supporting our final prediction, the numbers of animals adopted during the COLC were significantly lower than prior to the crisis for most animal types, but equines and guinea pigs were adopted at higher levels during the COLC.

Therefore, these findings do show concerning trends in the adoption rates of animals overall. These may reflect people’s decisions not to add to their cost of living by acquiring any or additional pets. Caution needs to be exercised when interpreting the findings of the increased adoption of equines and guinea pigs due to smaller numbers of animals, but it is interesting that these animals represent two extremes in terms of likely expenditure to maintain them. The RSPCA changed their approach to equine rehoming around this time, which could help explain this finding. There was more rehoming during the pandemic, as people decided that they had more time, and perhaps even money, available to take on a horse while there were restrictions. After the pandemic, the RSPCA shortened the probation period between horses going out to homes and being formally signed over. This likely improved the efficiency of rehoming during the pandemic and was carried through into post-pandemic times. Several factors have contributed to the decreasing numbers of equines being rehomed since the pandemic, including the loss of particular equine centres, the need for more staff at some centres and throughput being impacted by the need to house horses for longer in certain cases. It is worth highlighting here how much the RSPCA depends on private boarding to house the surplus of animals that cannot be housed at their sites. As this costs millions of pounds annually, it may signal a need for more space to be given over to such an important animal charity.

Cats and rabbits stayed waiting to be rehomed for longer during the pandemic than pre-pandemic and longer again post-pandemic, and it is noteworthy that the length of stay increased despite the numbers of cats and rabbits decreasing. Dogs also waited longer to be rehomed during the pandemic, but in contrast to the picture with cats and rabbits, the length of stay returned to pre-pandemic levels in the post-pandemic period.

### 4.3. Animal Returns

Over the three years of data that we were able to analyse (2018, 2021 and 2022), a downward trend in returns was observed for all animal types except rabbits, for which returns dropped during the pandemic but rose again post-pandemic. This supports our fourth prediction that following the pandemic, more animals would be taken in and/or returned because of the change in circumstances as people returned to work outside of the home. Through a comparison of the data from 2018, representing before the COLC, with 2022, during the crisis, a similar downward trend in the return of all animal types is clear, again not supporting our final prediction that the COLC would drive a higher return rate, and lending no direct support to the fear respondents expressed in the PAW Report [2] that the crisis would have a negative effect on pet welfare, at least not in terms of relinquishment.

For all animal types, fewer animals were returned within six months of having been adopted, which could be taken as encouraging in terms of owner persistence and commitment. We identified significant associations between each animal type and the reasons they were returned to the RSPCA. Dogs were more likely to be returned due to behaviour problems, followed by owners being unprepared for the commitment and a change in circumstances. Cats were returned more often due to behavioural issues, a change in circumstances and owners being unprepared. For equines, most returns were due to a change in circumstances. Rabbits were returned more often due to behavioural issues and a change in circumstances. Behavioural issues, a change in circumstances and owners being unprepared were also the main reasons why miscellaneous animals were returned and are in line with those identified previously [17,26]. That a change in circumstances was a common reason for returning animals lends some support to our first prediction and may be influenced by pressures created in owners’ work–life balance due to changes in working at home.

Through our use of ITS analysis, we have been able to provide an important summary of trends in the intake, adoption and return of companion animals in England and Wales. This can be used to show the impact of significant events such as the COVID-19 pandemic and the COLC and support organisations like the RSPCA in making predictions and decisions, informing policy and developing strategies to optimise the welfare of animals [32].

While we acknowledge that ITS analysis has some limitations, especially, in this case, in terms of explaining the reasons why the pandemic had the impact it did, our scope in terms of representing data from the whole of England and Wales means that the findings are generalisable and further research can use this same analysis to investigate trends in more stratified samples within the larger companion animal population. A further planned study into the reasons behind people’s decisions to adopt or return a companion animal will provide complimentary data to allow a bigger picture to become clear and to enable more individual-level inferences to be made from the data [36]. While another limitation of ITS is that the interruption should be the only change over time, and given that there could be a number of potential variables involved in the adoption and return of pets, we suggest that a global pandemic is a sufficiently unusual and large-scale disruption to overcome any threats to the validity of our approach.

This area of study is important within the context of human–animal interactions which, in turn, are a vital part of the bigger picture surrounding how all organisms (human and nonhuman) share space on a planet in jeopardy. More research like this is needed to address the crucial implications for the conservation of biodiversity and sustainability, including the fulfilment of the United Nations’ Sustainable Development Goals, including Life on Land and Good Health and Wellbeing, with these findings having important applications, especially in helping humans and animals to share space and help each other and in addressing the plight of homeless and abused animals. Just as nature-based solutions are proving powerful in the fight against climate change, e.g., by capturing more carbon in forests, investigating the power of companion animals in helping people’s wellbeing, especially during times of change, is key to understanding the importance of nonhuman pets and their role in a person’s life. We recommend further studies examining the power of pets in helping people and the plight of companion animals who need homing and rehoming, especially during times of uncertainty. Our study was opportunistic in examining the effects of a global pandemic and a crisis in terms of the cost of living, but it is also vital to carry out further research comparing the experiences of people with their pets during times of extreme stress and changes in lifestyle. Future research projects stemming from this are planned, including measuring differences in people’s psychological wellbeing and the benefit of companion animals over time and in situations that are pivotal in people’s lives. Extending the research to cover other situations in which people find their bonds with animals beneficial, such as during war, will build on this knowledge. Identifying what factors influence pet owners’ decisions to acquire a pet, and whether these factors are prevalent in different species and in different circumstances, might help our understanding of the outcomes people experience when living with their pet. It may also allow interventions to be planned with the aim of optimising compatibility at the adoption stage and reducing the relinquishment of companion animals.

Our analysis of intake and return rates suggests that the COLC does not appear to be forcing people to abandon or relinquish their companion animals due to financial burden. Although there were mixed results for the adoption data, most of the findings do suggest that adoption rates have decreased during the COLC. We examined data up to 2022; however, the effects of the COLC are still being felt up to the present day. We have provided forecast data and will compare these projections to data from 2023 onwards to determine if the COLC continues to have an impact on animals and pet ownership. It is possible that as the COLC continues, animals may be impacted more the longer people have been feeling the financial effects. Animal rescue organisations have not been immune to the effects of the COLC. They have also seen increasing costs post-pandemic, and although intake numbers fluctuate over the years, annual intake figures are still considerable. The additional burden placed on charities post-pandemic in terms of caring for cats and rabbits which are spending more time awaiting rehoming post-pandemic than pre-pandemic means that care provision is required for longer and that the additional length of stay impacts the ability of subsequent animals to be admitted. To continue taking in animals, throughput is crucial, so the rehoming of animals should be promoted as the primary choice for those looking to bring a new animal into their lives. Intake numbers are likely to remain sizable, so it is important for animals to be rehomed in a timely manner to allow for more animals to receive the help they require.

The findings from this study confirm what rescue centres have long known in regard to the seasonality of intake for cats and wildlife, for which they prepare each summer. In addition, we have now identified evidence of seasonality for rabbit and miscellaneous animal intake and for pet mammal, guinea pig, captive bird and equine adoptions. These trends, together with the forecasted predictions provided in this study, may help rescue centres to prepare accordingly. Pessimistically, it is possible that the world may experience more events like the pandemic, and therefore, the trends identified here can inform wide-scale readiness to optimise animal welfare at such times of human uncertainty. The proposed analysis of data from 2023 onwards may identify emerging post-pandemic trends which animal centres may find helpful to be aware of. It is important to highlight here too that our data support trends found in other studies from the US [14,15,22,23,24] which show that the media portrayal of huge increases in people adopting pets during the pandemic but also potentially returning pets afterwards, e.g., [13,20] has not been borne out.

We are very grateful for the opportunity to analyse data from the RSPCA, and cognisant of the plans for new data recording systems currently in the pipeline, we have some recommendations to offer. Ideally, it would be helpful to record data of animals being taken in, adopted and returned in as much detail as possible, although we realise that many of the staff running the branches are volunteers who are kept very busy and for whom record-keeping may not be seen as a priority. However, the more we know about the individual circumstances of ‘shifting’ animals, the better we can help them adapt and the more optimal the matching of individual animals with prospective individual owners may be. For example, there are likely to be differences in the numbers of dogs taken in depending on where they were purchased from, including from other rescue organisations and puppy mills or from online sources. The age and sex of the animals also likely play a role in the intake, adoption and relinquishment of animals. Those taken in due to cruelty or neglect may present different behavioural and possibly medical issues which would impact their rehoming and potentially increase the probability of return. That changes in circumstance and a lack of feeling prepared for the new pet were principal reasons for returning animals attests to the importance of this. More detailed information needs to be gathered on the specifics of these reasons, and owners returning pets need to be asked to what extent they researched what having such a pet would involve. In these ways, RSPCA staff will be much better able to target general campaigns and specific individual strategies to facilitate compatibility and a successful human–pet relationship. The consistent categorisation of animals across branches and centres, and across records of intake, adoption and return, would enable better comparisons over time and between sites. We recommend a whole-UK approach, similar to the national database maintained by Shelter Animals Count in the US, whereby records could be consistent across all rescue organisations, including the RSPCA. Ideally, recording the data in such a way as to enable the matching up of an individual animal’s journey (e.g., from intake and adoption to potential return) would allow a level of analysis that could directly highlight when and where problems occur and permit interventions to be made to optimise rehoming and minimise or prevent relinquishment. More detail on the specific changes in circumstances, on what new owners were not prepared for and on specific problematic behaviour issues would contribute greatly to the ability to tackle and reduce the relinquishment of animals.

In conclusion, pets have the power to prevent or at least mitigate much of the stress humans can experience in everyday life and during times of uncertainty, but these relationships are reciprocal and demand commitment from both sides.

## Figures and Tables

**Table 1:** Mean (SD) number of animals taken in by the RSPCA each month during the three time periods of the pandemic and the two time periods of the COLC.

Time Period									
	Pre-Pandemic			Pandemic			Post-Pandemic		
	Centres	Branches	Total	Centres	Branches	Total	Centres	Branches	Total
Animal Category									
Dog	267.56	670.75	938.31	156.31	375.06	531.38	198.63	489.88	688.50
	(50.38)	(115.62)	(141.05)	(49.57)	(88.77)	(125.12)	(49.90)	(346.80)	(81.61)
Cat	563.94	1959.31	2523.25	356.88	1397.63	1754.50	397.81	1508.75	1906.56
	(131.26)	(304.96)	(417.68)	(81.84)	(346.80)	(418.36)	(74.55)	(196.64)	(256.31)
Rabbit	80.38	207.44	287.81	51.88	164.31	216.19	80.19	168.25	248.44
	(19.96)	(30.28)	(40.68)	(32.92)	(49.72)	(76.23)	(33.78)	(24.97)	(43.57)
Wildlife	36.56	329.19	365.75	18.38	654.75	673.13	4.69	461.63	466.31
	(61.90)	(249.75)	(255.23)	(13.72)	(385.94)	(395.86)	(4.38)	(308.23)	(308.71)
Misc	221.75	388.56	610.31	291.63	327.63	619.25	144.06	287.06	431.13
	(72.13)	(239.81)	(251.34)	(389.40)	(172.83)	(470.12)	(47.80)	(58.83)	(83.01)
**Time Period**									
	**Pre-Cost-of-Living Crisis**			**Cost-of-Living Crisis**					
	Centres	Branches	Total	Centres	Branches	Total			
Animal Category									
Dog	210.35	521.38	731.74	200.57	488.86	689.43			
	(73.39)	(176.60)	(239.02)	(51.50)	(39.63)	(77.34)			
Cat	464.53	1681.56	2146.09	378.86	1477.00	1855.86			
	(147.10)	(417.19)	(550.57)	(55.59)	(186.39)	(230.82)			
Rabbit	64.85	185.38	250.24	85.29	166.93	252.21			
	(30.05)	(45.18)	(68.61	(32.84)	(24.25)	(44.92)			
Wildlife	26.15	513.88	540.03	4.64	404.07	408.71			
	(44.00)	(366.15)	(367.69)	(4.63)	(264.41)	(264.66)			
Misc	254.44	352.65	607.09	133.43	290.14	423.57			
	(269.58)	(203.06)	(361.47)	(36.85)	(59.94)	(78.82)			

Note: equines are not included here as they are not common to branches and centres.

**Table 2:** Interrupted time series analysis: model parameters for significant changes in animal intake.

Model Component	Estimate	t Value	*p* Value
*Closure*			
Dogs	−157.19	−2.078	0.044
*Pandemic*			
Cats	764.31	5.507	<0.001
Dogs	507.54	4.763	<0.001

**Table 3:** Mean (SD) number of animals adopted from the RSPCA each month during the three time periods of the pandemic and the two time periods of the COLC.

Time Period									
	Pre-Pandemic			Pandemic			Post-Pandemic		
	Centres	Branches	Total	Centres	Branches	Total	Centres	Branches	**Total**
Animal Category									
Dog	84.56	588.88	673.44	67.75	319.75	387.50	74.50	411.38	**485.88**
	(32.74)	(129.65)	(117.15)	(22.13)	(72.07)	(89.09)	(12.45)	(34.48)	**(36.92)**
Cat	294.94	1731.75	2026.69	195.56	1201.31	1396.88	197.94	1338.00	**1535.94**
	(108.15)	(352.37)	(395.27)	(57.97)	(342.48)	(392.95)	(34.32)	(209.95)	**(238.72)**
Rabbit	40.00	181.00	221.00	31.75	139.00	170.75	25.81	146.75	**172.56**
	(17.76)	(19.45)	(27.27)	(12.40)	(28.34)	(36.94)	(8.87)	(16.06)	**(18.68)**
**Time Period**									
	**Pre-Cost-of-Living Crisis**			**Cost-of-Living Crisis**					
	Centres	Branches	Total	Centres	Branches	Total			
Animal Category									
Dog	76.06	451.40	527.46	74.38	409.31	483.69			
	(27.63)	(164.04)	(170.10)	(27.63)	(35.73)	(39.53)			
Cat	241.94	1468.49	1710.43	195.92	1303.08	1499.00			
	(95.64)	(416.07)	(480.62)	(35.76)	(216.99)	(248.36)			
Rabbit	34.94	160.06	195.00	26.00	143.54	169.54			
	(15.37)	(30.64)	(39.21)	(9.38)	(15.99)	(19.25)			

Note: animal types not common to centres and branches do not appear here.

**Table 4:** Interrupted time series analysis: model parameters for significant changes in animal adoption.

Model Component	Estimate	*t* Value	*p* Value
*Closure*			
Cats	−519.92	−3.662	<0.001
Rabbits	−57.40	−2.75	0.009
*Pandemic*			
Cats	997.30	4.976	<0.001
Dogs	494	4.767	<0.001

**Table 5:** Length of stay (days) in RSPCA animal centres over time for cats.

Time Period	Cats (*n*)	Mean	SD
Pre-pandemic	5468	59.13	50.84
Pandemic	3132	77.49	83.03
Post-pandemic	3168	85.36	92.07

**Table 6:** Length of stay (days) in RSPCA animal centres over time for dogs.

Time Period	*n*	Mean	SD
Pre-pandemic	1970	150.36	171.07
Pandemic	1139	180.80	231.41
Post-pandemic	1200	150.42	202.83

**Table 7:** Length of stay (days) in RSPCA animal centres over time for rabbits.

Time Period	*n*	Mean	SD
Pre-pandemic	747	76.34	67.14
Pandemic	508	119.28	93.34
Post-pandemic	413	154.77	122.83

**Table 8:** Total animals returned within six months and after six months.

Year	Within Six Months	After Six Months
2018	344	518
2021	190	269
2022	149	235
Total	683	1022

## Data Availability

Data are available from the corresponding author upon request.

## References

[1] Global Pets Survey. https://globalpetindustry.com/news/global-pet-population-at-1-billion-cats-lead-the-way/.

[2] (2023). PAW Report. https://www.pdsa.org.uk/what-we-do/pdsa-animal-wellbeing-report/paw-report-2023.

[3] Wensley S., Betton V., Gosschalk K., Hooker R., Main D., Martin N., Tipton E. (2021). Driving evidence-based improvements for the UK’s ‘Stressed. Lonely. Overweight. Bored. Aggressive. Misunderstood…but loved’ companion animals. Vet. Rec..

[4] Gan G.Z.H., Hill A., Carryer J., Keesing S., Netto J. (2019). Pet ownership and its influence on mental health in older adults. Aging Ment. Health.

[5] Mein G., Grant R. (2018). A cross-sectional exploratory analysis between pet ownership, sleep, exercise, health and neighbourhood perceptions: The Whitehall II cohort study. BMC Geriatr..

[6] Plante A., Bedrossian N., Cadotte G., Piché A., Michael F., Bédard S., Tessier H., Fernandez-Prada C., Sabiston C., Dieudé M. (2013). Pet ownership and lifestyle behaviours of immunosuppressed individuals and their relatives in the context of COVID-19 pandemic. Prev. Med. Rep..

[7] Tinbergen N. (1963). On aims and methods of ethology. Z. Tierpsychol..

[8] Pongrácz P., Dobos P. (2023). What is a companion animal? An ethological approach based on Tinbergen’s four questions. Critical review. Appl. Anim. Behav. Sci..

[9] Scoresby K.J., Strand E.B., Ng Z., Brown K.C., Stilz C.R., Strobel K., Barroso C.S., Souza M. (2021). Pet ownership and Quality of Life: A systematic review of the literature. Vet. Sci..

[10] Hargreaves E.A., Lee C., Jenkins M., Calverley J.R., Hodge K., Houge Mackenzie S. (2021). Changes in physical activity pre-, during and post-lockdown COVID-19 restrictions in New Zealand and the explanatory role of daily hassles. Front. Psychol..

[11] Ho J., Hussain S., Sparagano O. (2021). Did the COVID-19 pandemic spark a public interest in pet adoption?. Front. Vet. Sci..

[12] Tan J.S.Q., Fung W., Tan BS W., Low J.Y., Syn N.L., Goh Y.X., Pang J. (2021). Association between pet ownership and physical activity and mental health during the COVID-19 “circuit breaker” in Singapore. One Health.

[13] Barr S. (2020). Coronavirus Pandemic Sees Huge Increase in Cat and Dog Adoptions. https://www.independent.co.uk/life-style/coronavirus-dog-cat-pet-adoption-battersea-rehome-covid-19-a9426741.html.

[14] Hoffman C.L., Thibault M., Hong J. (2021). Characterizing Pet Acquisition and Retention During the COVID-19 Pandemic. Front. Vet. Sci..

[15] Powell L., Houlihan C., Stone M., Gitlin I., Ji X., Reinhard C.L., Watson B. (2020). Animal shelters’ response to the COVID-19 pandemic: A pilot survey of 14 shelters in the Northeastern United States. Animals.

[16] Powell L., Reinhard C., Satriale D., Morris M., Serpell J., Watson B. (2021). Characterizing unsuccessful animal adoptions: Age and breed predict the likelihood of return, reasons for return and post-return outcomes. Sci. Rep..

[17] Powell L., Reinhard C.L., Satriale D., Morris M., Serpell J., Watson B. (2022). The impact of returning a pet to the shelter on future animal adoptions. Sci. Rep..

[18] Amiot C.E., Sukhanova K., Bastian B. (2020). Social identification with animals: Unpacking our psychological connection with other animals. J. Personal. Soc. Psychol..

[19] Carroll G.A., Torjussen A., Reeve C. (2023). Companion animal adoption and relinquishment during the COVID-19 pandemic: Households with children at greatest risk of relinquishing a cat or dog. Anim. Welf..

[20] Johnson R., Sherlock G. (2025). Dogs Given Up Since Pandemic at ‘Record High’. https://www.bbc.co.uk/news/articles/cy8pejl63qqo.

[21] Morgan L., Protopopova A., Birkler R.I.D., Itin-Shwartz B., Sutton G.A., Gamliel A., Yakobson B. (2020). Human–dog relationships during the COVID-19 pandemic: Booming dog adoption during social isolation. Humanit. Soc. Sci. Commun..

[22] American Society for the Prevention of Cruelty to Animals (2021). New ASPCA Survey Shows Overwhelming Majority of Dogs and Cats Acquired During the Pandemic Are Still in Their Homes. https://www.aspca.org/about-us/press-releases/new-aspca-survey-shows-overwhelming-majority-dogs-and-cats-acquired-during.

[23] Doherty C. (2022). No shelter: What the data say on animal relinquishments. Can. Vet. J..

[24] Shelter Animals Count (2025). Intake and Outcome Database. https://www.shelteranimalscount.org/about-the-data/.

[25] Holland K.E. (2019). Acquiring a pet dog: A review of factors affecting the decision-making of prospective dog owners. Animals.

[26] Lambert K., Coe J., Niel L., Dewey C., Sargeant J. (2015). A systematic review and meta- analysis of the proportion of dogs surrendered for dog-related and owner-related reasons. Prev. Vet. Med..

[27] Buller K., Ballantyne K. (2020). Living with and loving a pet with behavioral problems: Pet owners’ experiences. J. Vet. Behav..

[28] Hoefman R., Payakachat N., van Exel J., Kuhlthau K., Kovacs E., Pyne J., Tilford J. (2014). Caring for a child with autism spectrum disorder and parents’ quality of life: Application of the CarerQol. J. Autism Dev. Disord..

[29] Stone R., Lonnie M., Brown A., Douglas F., Johnstone M., Green M., Hardman C., Hunter E., on behalf of the FIO-Food Team (2024). The impact of the cost of living crisis and food insecurity on food purchasing behaviours and food preparation practices in people living with obesity. Appetite.

[30] Sangha M., Baker M., Baldwin A., Murray A. (2025). Assessing the effect of the cost-of living crisis on hot water bottle-related burns in the United Kingdom, a single-centre retrospective observational study. JPRAS Open.

[31] Bernal J.L., Cummins S., Gasparrini A. (2017). Interrupted time series regression for the evaluation of public health interventions: A tutorial. Int. J. Epidemiol..

[32] Penfold R., Zhang F. (2013). Use of interrupted time series analysis in evaluating health care quality improvements. Acad. Pediatr..

[33] Zhang Y., Ren Y., Huang Y., Yao M., Jia Y., Wang Y., Mei F., Zou K., Tan J., Sun X. (2024). Design and statistical analysis reporting among interrupted time series studies in drug utilization research: A cross-sectional survey. BMC Med. Res. Methodol..

[34] Wagner A.K., Soumerai S.B., Zhang F., Ross-Degnan D. (2002). Segmented regression analysis of interrupted time series studies in medication use research. J. Clin. Pharm. Ther..

[35] Karlinsky A., Kobak D. (2021). Tracking excess mortality across countries during the COVID-19 pandemic with the World Mortality Dataset. eLife.

[36] Hanbury A., Farley K., Thompson C., Wilson P.M., Chambers D., Holmes H. (2013). Immediate versus sustained effects: Interrupted time series analysis of a tailored intervention. Implement. Sci..

